# Supplementary material for: Aci-bench: a Novel Ambient Clinical Intelligence Dataset for Benchmarking Automatic Visit Note Generation
Source: Sci Data. 2023 Sep 6;10:586. doi: 10.1038/s41597-023-02487-3 (PMC10482860; doi:10.1038/s41597-023-02487-3)
Supplement: Supplementary file 1 — Supplementary Information [file 41597_2023_2487_MOESM1_ESM.pdf]

# Supplementary Information

## Contents


1 OpenAI post-processing 1
2 Sample model outputs 1
3 Baseline hyper-parameters 2
4 Supplementary Results 3


## 1 OpenAI post-processing

| models | description |
|---|---|
| ChatGpt | `text = text.replace('\n', ' ').strip()`<br>`for division in [ 'HISTORY OF PRESENT ILLNESS', 'PHYSICAL EXAM', 'RESULTS', 'ASSESSMENT AND PLAN' ] :`<br>`text = text.replace( '%s' %division , '\n%s\n' %division )`<br>`text = text.replace( '<%s>' \%division , '\n%s\n' %division )`<br>`text = text.replace( '%s:'\%division , '\n%s\n' %division )`<br>`text = text.replace( '# %s:'\%division , '\n%s\n' %division )`<br>`text = text.replace( '# %s'\%division , '\n%s\n' %division )`<br>`text = text.replace( '## %s'\%division , '\n%s\n' %division )`<br>`text = text.replace( '**%s**'\%division , '\n%s\n' %division )` |
| Text-Davinci-002,Text-Davinci-003 | `text.replace('\n', ' ').strip().replace( 'PHYSICAL EXAM:', '\nPHYSICAL EXAM:\n' )`<br>`.replace( 'RESULTS:', '\nRESULTS:\n' )`<br>`.replace( 'ASSESSMENT AND PLAN:', '\nASSESSMENT AND PLAN:\n' )` |
| GPT-4 | `text = text.replace('\n', ' ').strip().`<br>`if (text.startswith("Possible summary:") or`<br>`text.startswith("Possible clinical note:") or`<br>`text.startswith("A possible clinical note is:")) :`<br>`text = text[text.index(":")+1:]`<br>`text.replace( 'PHYSICAL EXAM:', '\nPHYSICAL EXAM:\n' )`<br>`.replace( 'RESULTS:', '\nRESULTS:\n' )`<br>`.replace( 'ASSESSMENT AND PLAN:', '\nASSESSMENT AND PLAN:\n' )` |

**Table S1.** Open-AI post-processing rules. In order to ensure the rule-based section algorithm may correctly split into divisions, we added several simple post-processing rules tailored to the algorithm.

## 2 Sample model outputs

We investigated example outputs from different models, to generate notes from the transcript *D2N080* in the validation set. As demonstrated in Table S2, both BART+FT$_{\text{SAMSum}}$ (Division) and GPT-4 excelled at condensing dialogue information into a coherent clinical note. However, among all the models, only GPT-4 properly identified the patient's correction on the doctor's mistake in the transcript from “right knee pain" to “left knee pain". Meanwhile, BART+FT$_{\text{SAMSum}}$ (Division) only missed crucial pain-related information and instead focused on less important details about the patient's travel.

| **Transcript** | | **Note** |
| --- | --- | --- |
| ...<br>[doctor] i <understand> you’re you’ve come in with some <right knee pain> can you tell me about it what’s going on<br>[patient] it it’s not the <right knee> it’s the <left knee><br>[doctor] okay the **<left knee>**<br>[patient] and it just happens occasionally less than once a day when i’m <walking> all of a sudden it is kind of <like> gives out and i <think> here i’m going to <fall> but i usually <catch> myself so <lot> of times i have to hold a grocery cart and that helps a <lot> so it comes and goes and it it passes just about as quickly as it comes i do n’t know what it is whether i stepped wrong or i just don’t know... | | <CHIEF COMPLAINT> **<Left knee pain>**.<br><HISTORY OF PRESENT ILLNESS><br>Andrea Barnes is a 34-year-old <female> who presents today for evaluation of <left knee pain>. The patient has been experiencing intermittent episodes of <pain> and sudden instability with ambulation. Her <pain> is localized deep in her <patella> and occurs less than once daily... |
| **Prediction** | | |
| **train$_{\text{UMLS}}$** | **BART+FT$_{\text{SAMSum}}$** | **BART+FT$_{\text{SAMSum}}$ (Division)** |
| <CHIEF COMPLAINT> **Annual exam**.<br><HISTORY OF PRESENT ILLNESS><br>Martha Collins is a 50-year-old <female> with a <past medical history> significant for <congestive heart failure>, <depression>,and <hypertension> who presents for her annual exam. It has been a year since I last <saw> the patient... | <CHIEF COMPLAINT> **<Right knee pain>**.<br><HISTORY OF PRESENT ILLNESS><br>The patient reports she has <right knee pain>, which she <experiences> occasionally less than once a day when she is <walking>. She does not know the cause of the <pain>. She denies any specific <injury> to her <knee>... | <CHIEF COMPLAINT> **<Right knee pain>**.<br><SOCIAL HISTORY><br><Patient> reports she <likes> to travel. She has a <trip> planned for the next month in which she will <probably> be doing an <lot> of <walking>. She is also <planning> to attend a lion’s club <function> in which there are line dances. She does not <feel> her <knee> is in good <condition> for this type of activity She denies <hearing> or <feeling> a pop at any point... |
| **Prediction** | | |
| **LED+FT$_{\text{PubMed}}$** | **LED+FT$_{\text{PubMed}}$ (Division)** | **GPT-4** |
| **CHKnee <pain>** is a common <complaint> in the <emergency> room today. The patient reports that it occurs less frequently than previously thought and she does not know if her <symptoms> are caused by <walking> on <unstable> surfaces or due to an underlying <inflammatory disease> such as <diabetes mellitus>, <hypercholesterolemia> (hcm)... | CHHISTORY OF <PRESENT> ILLNESS<br>Andrea <Diaz> is a pleasant 29-year old <female> who presents to the clinic today for evaluation of **<right knee pain>**. The onset and severity are <unknown> but they usually resolve themselves quickly on their own without treatment or therapy... | <HISTORY OF PRESENT ILLNESS>: The patient is a 65-year-old <female> who presents with intermittent **<left knee pain>** that occurs less than once a day when <walking>. She describes it as a <feeling> of the <knee> giving out and the kneecap fading. She denies any <trauma>, pop, or <swelling>. She has not taken any <analgesics>. She is concerned about her upcoming <trip> that involves <walking> and line <dancing>... |

**Table S2.** Example outputs from different models, to generate notes from the transcript D2N080 in the validation set (reformatted). The UMLS concepts detected in fact-based evaluation are included inside angle brackets.

## 3 Baseline hyper-parameters

The fine-tuning and note generation hyper-parameters for BART- and LED-based baseline models can be found in Table S3. Note that the max target token length is smaller than the total length of clinical notes. Because the BART- and LED-based baseline models are not initially pretrained with such a long token length as clinical notes, a longer max target token length does not have a very good generation result from our experiment.

| Hyper-parameter | BART-Based | LED-Based |
| --- | --- | --- |
| Max source token length | 1024 | 2048 |
| Max target token length | 256 | 256 |
| Min target token length | 128 | 128 |
| Batch size | 1 | 2 |
| Epochs | 10 | 15 |
| Learning Rate | $10^{-5}$ | $10^{-5}$ |
| Weight decay | 0 | 0 |
| Beam size | 5 | 5 |
| Global attention | - | 128 |

**Table S3.** The fine-tuning and note generation hyper-parameters for BART- and LED-based baseline models.

## 4 Supplementary Results

The following tables offer results on test2 and test3 full note and division-based results for comparison.

| Model | ROUGE-1 | ROUGE-2 | ROUGE-L | MEDCON |
|---|---|---|---|---|
| **Transcript-copy-and-paste** | | | | |
| longest spearker turn | 28.96 | 10.43 | 24.46 | 34.30 |
| longest doctor turn | 28.96 | 10.43 | 24.46 | 34.30 |
| 12 speaker turns | 31.57 | 10.59 | 28.49 | 33.53 |
| 12 doctor turns | 37.89 | 14.01 | 34.63 | 50.12 |
| transcript | 32.34 | 13.07 | 30.32 | 55.38 |
| **Retrieval-based** | | | | |
| $\text{train}_{\text{UMLS}}$ | 44.41 | 17.66 | 40.81 | 35.67 |
| $\text{train}_{\text{sent}}$ | 44.10 | 16.68 | 40.40 | 27.66 |
| **BART-based** | | | | |
| BART | 41.90 | 19.87 | 34.56 | 44.39 |
| BART (Division) | **52.63** | **24.53** | 46.71 | 46.97 |
| $\text{BART+FT}_{\text{SAMSum}}$ | 40.37 | 18.86 | 34.26 | 44.17 |
| $\text{BART+FT}_{\text{SAMSum}}$ (Division) | 52.08 | 24.37 | **47.16** | 48.12 |
| BioBART | 39.00 | 18.44 | 33.40 | 43.05 |
| BioBART (Division) | 50.80 | 22.70 | 46.13 | 44.76 |
| **LED-based** | | | | |
| LED | 29.40 | 6.50 | 23.61 | 32.65 |
| LED (Division) | 35.14 | 8.57 | 30.84 | 34.24 |
| $\text{LED+FT}_{\text{PubMed}}$ | 27.66 | 6.13 | 22.31 | 31.98 |
| $\text{LED+FT}_{\text{PubMed}}$ (Division) | 31.21 | 7.37 | 27.60 | 32.74 |
| **OpenAI (wo FT)** | | | | |
| Text-Davinci-002 | 39.36 | 16.95 | 36.15 | 46.47 |
| Text-Davinci-003 | 43.65 | 21.21 | 40.59 | **55.92** |
| ChatGPT | 42.30 | 16.57 | 37.31 | 49.50 |
| GPT-4 | 51.24 | 21.65 | 45.60 | 55.04 |

**Table S4.** Results of the summarization models at the full note level, test set 2. As in test 1, the most competitive models are the division-based BART models and GPT-4 in terms of Rouge scores. Unlike test 1, Text-Davinci-003 had a higher MEDCON performance than GPT4.

| Model | Evaluation score on the SUBJECTIVE division | | | | | | |
|---|---|---|---|---|---|---|---|
| | ROUGE-1 | ROUGE-2 | ROUGE-L | BERTScore | BLEURT | MEDCON | Average |
| **Retrieval-based** | | | | | | | |
| $\text{train}_{\text{UMLS}}$ | 41.99 | 22.78 | 31.46 | 71.30 | 39.60 | 23.56 | 41.63 |
| $\text{train}_{\text{sent}}$ | 43.56 | 22.34 | 31.45 | 71.23 | 37.83 | 18.02 | 39.88 |
| **BART-based** | | | | | | | |
| BART | 47.79 | 26.91 | 29.42 | 68.40 | 44.40 | **47.93** | 48.86 |
| BART (Division) | 50.41 | 28.98 | 32.72 | 70.14 | 45.44 | 47.40 | 50.09 |
| $\text{BART+FT}_{\text{SAMSum}}$ | 46.74 | 25.99 | 30.19 | 68.70 | **45.76** | 45.83 | 48.65 |
| $\text{BART+FT}_{\text{SAMSum}}$ (Division) | **50.43** | **30.11** | 35.10 | **71.79** | 45.05 | 46.61 | **50.50** |
| BioBART | 48.37 | 26.72 | 31.47 | 69.25 | 43.18 | 45.36 | 48.33 |
| BioBART (Division) | 48.99 | 28.90 | **35.14** | 71.21 | 43.76 | 44.61 | 49.31 |
| **LED-based** | | | | | | | |
| LED | 25.00 | 6.05 | 11.12 | 55.56 | 29.96 | 22.25 | 30.46 |
| LED (Division) | 31.21 | 8.55 | 16.12 | 57.06 | 28.08 | 24.21 | 31.99 |
| $\text{LED+FT}_{\text{PubMed}}$ | 23.20 | 5.37 | 10.50 | 54.97 | 22.04 | 19.20 | 27.31 |
| $\text{LED+FT}_{\text{PubMed}}$ (Division) | 25.37 | 6.69 | 12.79 | 56.18 | 19.67 | 20.99 | 27.95 |
| **OpenAI (wo FT)** | | | | | | | |
| Text-Davinci-002 | 27.11 | 13.01 | 19.81 | 56.48 | 36.22 | 31.74 | 36.10 |
| Text-Davinci-003 | 29.05 | 15.00 | 22.88 | 58.97 | 37.40 | 38.32 | 39.25 |
| ChatGPT | 27.65 | 12.40 | 18.92 | 59.55 | 38.37 | 32.17 | 37.44 |
| GPT-4 | 40.40 | 18.72 | 26.81 | 63.13 | 43.59 | 46.98 | 45.58 |

**Table S5.** Results of the summarization models on the SUBJECTIVE division, test set 2. Similar to test 1, the best overall model proved to be $\text{BART+FT}_{\text{SAMSum}}$.

| Model | Evaluation score on the OBJECTIVE_EXAM division | | | | | | |
|---|---|---|---|---|---|---|---|
| | ROUGE-1 | ROUGE-2 | ROUGE-L | BERTScore | BLEURT | MEDCON | Average |
| **Retrieval-based** | | | | | | | |
| $\text{train}_{\text{UMLS}}$ | 44.48 | 26.70 | 36.60 | **73.08** | 40.80 | 23.52 | 43.33 |
| $\text{train}_{\text{sent}}$ | 41.14 | 21.58 | 33.60 | 72.43 | 38.35 | 18.07 | 40.24 |
| **BART-based** | | | | | | | |
| BART | 2.99 | 0.07 | 2.99 | 40.87 | 14.28 | 0.00 | 14.29 |
| BART (Division) | **48.36** | **28.19** | 35.29 | 71.94 | 41.75 | 32.55 | 45.88 |
| $\text{BART+FT}_{\text{SAMSum}}$ | 2.83 | 0.26 | 2.83 | 40.53 | 14.36 | 0.00 | 14.22 |
| $\text{BART+FT}_{\text{SAMSum}}$ (Division) | 46.52 | 28.00 | 34.87 | 71.81 | 40.63 | 31.94 | 45.21 |
| BioBART | 0.00 | 0.00 | 0.00 | 0.00 | 17.28 | 0.00 | 4.32 |
| BioBART (Division) | 42.31 | 23.90 | 30.42 | 70.45 | 39.43 | 28.18 | 42.57 |
| **LED-based** | | | | | | | |
| LED | 0.00 | 0.00 | 0.00 | 0.00 | 17.28 | 0.00 | 4.32 |
| LED (Division) | 27.79 | 7.87 | 16.16 | 54.41 | 15.00 | 20.75 | 26.86 |
| $\text{LED+FT}_{\text{PubMed}}$ | 0.00 | 0.00 | 0.00 | 0.00 | 17.28 | 0.00 | 4.32 |
| $\text{LED+FT}_{\text{PubMed}}$ (Division) | 21.05 | 5.85 | 11.53 | 54.01 | 13.99 | 16.46 | 24.32 |
| **OpenAI (wo FT)** | | | | | | | |
| Text-Davinci-002 | 38.73 | 19.19 | 30.53 | 65.51 | 43.69 | 39.93 | 44.65 |
| Text-Davinci-003 | 47.30 | 27.30 | **37.70** | 69.44 | 47.69 | **47.76** | **50.58** |
| ChatGPT | 30.67 | 12.69 | 24.88 | 59.88 | 36.26 | 28.79 | 36.92 |
| GPT-4 | 45.55 | 23.17 | 36.61 | 69.11 | **49.13** | 46.31 | 49.91 |

**Table S6.** Results of the summarization models on the OBJECTIVE_EXAM division, test set 2. Similar to test 1, LED models found this division challenging to summarize with full note and the most performant model based on the averaged score is Text-davinci-003 with BART (Division) second best.

| Model | Evaluation score on the OBJECTIVE_RESULTS division | | | | | | |
|---|---|---|---|---|---|---|---|
| | ROUGE-1 | ROUGE-2 | ROUGE-L | BERTScore | BLEURT | MEDCON | Average |
| **Retrieval-based** | | | | | | | |
| $train_{UMLS}$ | **40.09** | 14.85 | **39.62** | 71.37 | 46.40 | 13.13 | 40.61 |
| $train_{sent}$ | 39.18 | 16.08 | 38.44 | **72.69** | 43.36 | 7.25 | 38.63 |
| **BART-based** | | | | | | | |
| BART | 22.50 | 0.00 | 22.50 | 56.07 | 30.48 | 0.00 | 25.39 |
| BART (Division) | 25.52 | 15.65 | 23.47 | 63.19 | 39.47 | 21.35 | 36.39 |
| BART+$FT_{SAMSum}$ | 0.00 | 0.00 | 0.00 | 0.00 | 6.95 | 0.00 | 1.74 |
| BART+$FT_{SAMSum}$ (Division) | 22.97 | 13.74 | 21.27 | 62.14 | 37.45 | 15.34 | 33.56 |
| BioBART | 0.00 | 0.00 | 0.00 | 0.00 | 6.95 | 0.00 | 1.74 |
| BioBART (Division) | 23.38 | 12.66 | 21.88 | 61.99 | 39.44 | 16.35 | 34.27 |
| **LED-based** | | | | | | | |
| LED | 0.00 | 0.00 | 0.00 | 0.00 | 6.95 | 0.00 | 1.74 |
| LED (Division) | 12.31 | 4.26 | 9.58 | 47.64 | 9.08 | 7.31 | 18.19 |
| LED+$FT_{PubMed}$ | 0.00 | 0.00 | 0.00 | 0.00 | 6.95 | 0.00 | 1.74 |
| LED+$FT_{PubMed}$ (Division) | 8.85 | 2.88 | 7.26 | 42.02 | 6.30 | 7.63 | 15.57 |
| **OpenAI (wo FT)** | | | | | | | |
| Text-Davinci-002 | 34.17 | 15.76 | 32.82 | 67.08 | 47.62 | 19.25 | 40.38 |
| Text-Davinci-003 | 36.73 | **20.71** | 36.12 | 67.73 | **49.28** | 22.64 | **42.71** |
| ChatGPT | 25.92 | 6.86 | 25.20 | 60.72 | 39.16 | 9.45 | 32.16 |
| GPT-4 | 32.18 | 16.86 | 29.71 | 64.82 | 47.13 | **25.24** | 40.86 |

**Table S7.** Results of the summarization models on the OBJECTIVE_RESULTS division, test set 2. Similar OBJECTIVE_EXAM results, LED models performed suboptimally for the objective_results division. This could be as a result of minimal information content for this division (often empty) as well as this content appearing later in a long sequence. OpenAI models performed the best in this division.

| Model | Evaluation score on the ASSESSMENT_AND_PLAN division | | | | | | |
|---|---|---|---|---|---|---|---|
| | ROUGE-1 | ROUGE-2 | ROUGE-L | BERTScore | BLEURT | MEDCON | Average |
| **Retrieval-based** | | | | | | | |
| $train_{UMLS}$ | **45.13** | **21.24** | **29.65** | **70.96** | 43.72 | 25.30 | 43.00 |
| $train_{sent}$ | 42.10 | 20.02 | 28.27 | 70.09 | 42.56 | 16.93 | 39.93 |
| **BART-based** | | | | | | | |
| BART | 0.79 | 0.26 | 0.57 | 34.86 | 19.88 | 0.00 | 13.82 |
| BART (Division) | 42.70 | 19.47 | 25.00 | 67.25 | 40.51 | 30.79 | 41.90 |
| BART+$FT_{SAMSum}$ | 1.19 | 0.45 | 0.65 | 35.07 | 20.20 | 0.54 | 14.14 |
| BART+$FT_{SAMSum}$ (Division) | 42.59 | 19.58 | 25.61 | 67.66 | 41.12 | 32.22 | 42.56 |
| BioBART | 0.48 | 0.14 | 0.34 | 34.64 | 19.27 | 0.62 | 13.71 |
| BioBART (Division) | 41.96 | 19.05 | 25.59 | 66.95 | 41.15 | 28.58 | 41.39 |
| **LED-based** | | | | | | | |
| LED | 0.00 | 0.00 | 0.00 | 0.00 | 29.99 | 0.00 | 7.50 |
| LED (Division) | 28.96 | 6.08 | 12.53 | 56.30 | 28.09 | 22.51 | 30.69 |
| LED+$FT_{PubMed}$ | 0.00 | 0.00 | 0.00 | 0.00 | 29.99 | 0.00 | 7.50 |
| LED+$FT_{PubMed}$ (Division) | 29.47 | 6.33 | 12.80 | 55.98 | 21.21 | 24.29 | 29.42 |
| **OpenAI (wo FT)** | | | | | | | |
| Text-Davinci-002 | 30.66 | 11.84 | 19.38 | 59.95 | 44.54 | 34.33 | 39.86 |
| Text-Davinci-003 | 34.91 | 15.88 | 25.06 | 63.25 | 47.74 | **41.50** | 44.44 |
| ChatGPT | 25.22 | 9.20 | 15.75 | 54.75 | 40.81 | 27.93 | 35.05 |
| GPT-4 | 39.38 | 15.22 | 24.76 | 64.56 | **49.78** | 38.67 | **44.86** |

**Table S8.** Results of the summarization models on the ASSESSMENT_AND_PLAN, test set 2. BART and LED models trained for full note generation perform suboptimally, likely as this content appearing later in a long sequence. Similar to test 1, the best models for ASSESSMENT_AND_PLAN are the BART Division models and the OpenAI Text-davinci-003 and GPT4 models.

| **Model** | **ROUGE-1** | **ROUGE-2** | **ROUGE-L** | **MEDCON** |
|---|---|---|---|---|
| **Transcript-copy-and-paste** | | | | |
| longest spearker turn | 25.37 | 9.05 | 21.85 | 29.30 |
| longest doctor turn | 25.13 | 9.04 | 21.79 | 29.60 |
| 12 speaker turns | 31.00 | 10.72 | 28.68 | 36.60 |
| 12 doctor turns | 34.69 | 12.51 | 32.27 | 45.42 |
| transcript | 32.75 | 12.78 | 30.91 | **56.31** |
| **Retrieval-based** | | | | |
| $\text{train}_{\text{UMLS}}$ | 48.00 | 20.57 | 44.61 | 38.99 |
| $\text{train}_{\text{sent}}$ | 40.86 | 14.66 | 37.64 | 25.24 |
| **BART-based** | | | | |
| BART | 40.54 | 18.52 | 34.62 | 44.92 |
| BART (Division) | 51.79 | 23.34 | 46.62 | 46.06 |
| $\text{BART+FT}_{\text{SAMSum}}$ | 39.38 | 18.38 | 33.89 | 46.01 |
| $\text{BART+FT}_{\text{SAMSum}}$ (Division) | **52.77** | **24.38** | **48.03** | 47.56 |
| BioBART | 38.32 | 17.39 | 33.39 | 43.06 |
| BioBART (Division) | 50.28 | 22.95 | 46.09 | 43.21 |
| **LED-based** | | | | |
| LED | 28.96 | 5.80 | 23.66 | 33.47 |
| LED (Division) | 34.71 | 8.03 | 30.77 | 33.79 |
| $\text{LED+FT}_{\text{PubMed}}$ | 26.32 | 5.24 | 21.92 | 27.53 |
| $\text{LED+FT}_{\text{PubMed}}$ (Division) | 31.07 | 7.52 | 27.83 | 33.74 |
| **OpenAI (wo FT)** | | | | |
| Text-Davinci-002 | 41.02 | 18.93 | 38.50 | 49.05 |
| Text-Davinci-003 | 42.57 | 21.13 | 39.89 | 54.93 |
| ChatGPT | 46.08 | 19.36 | 41.72 | 52.47 |
| GPT-4 | 50.30 | 21.67 | 45.67 | 54.98 |

**Table S9.** Results of the summarization models at the full note level, test set 3. As in test 1, the most competitive models are the division-based BART models and GPT-4 in terms of Rouge scores. Unlike test 1, the trancript-copy baseline had a highest MEDCON performance than GPT4. However still the OpenAI models were the next best models in terms of both Rouge and MEDCON scores.

| Model | Evaluation score on the SUBJECTIVE division | | | | | | |
|---|---|---|---|---|---|---|---|
| | ROUGE-1 | ROUGE-2 | ROUGE-L | BERTScore | BLEURT | MEDCON | Average |
| **Retrieval-based** | | | | | | | |
| $\text{train}_{\text{UMLS}}$ | 47.49 | 26.86 | 35.45 | **73.83** | 40.89 | 26.80 | 44.53 |
| $\text{train}_{\text{sent}}$ | 41.20 | 21.42 | 30.18 | 71.08 | 39.07 | 15.27 | 39.09 |
| **BART-based** | | | | | | | |
| BART | 47.02 | 24.99 | 28.86 | 68.01 | 43.75 | 43.59 | 47.24 |
| BART (Division) | 48.92 | 27.14 | 31.77 | 70.15 | **44.75** | 45.12 | 48.99 |
| $\text{BART+FT}_{\text{SAMSum}}$ | 46.96 | 25.11 | 30.06 | 69.36 | 44.18 | 44.15 | 47.93 |
| $\text{BART+FT}_{\text{SAMSum}}$ (Division) | **52.35** | **29.96** | **35.60** | 72.23 | 43.02 | **45.77** | **50.08** |
| BioBART | 46.77 | 24.70 | 30.13 | 68.68 | 42.06 | 39.58 | 46.05 |
| BioBART (Division) | 47.51 | 26.50 | 32.83 | 70.52 | 42.99 | 38.37 | 46.87 |
| **LED-based** | | | | | | | |
| LED | 24.42 | 5.57 | 11.19 | 55.48 | 29.34 | 20.76 | 29.83 |
| LED (Division) | 31.28 | 8.59 | 16.08 | 57.59 | 27.66 | 24.15 | 32.01 |
| $\text{LED+FT}_{\text{PubMed}}$ | 22.47 | 4.99 | 10.07 | 54.53 | 20.13 | 16.35 | 25.88 |
| $\text{LED+FT}_{\text{PubMed}}$ (Division) | 25.10 | 6.56 | 12.68 | 56.36 | 19.57 | 19.43 | 27.54 |
| **OpenAI (wo FT)** | | | | | | | |
| Text-Davinci-002 | 29.09 | 13.13 | 20.66 | 57.86 | 37.65 | 34.41 | 37.72 |
| Text-Davinci-003 | 29.97 | 14.75 | 22.17 | 58.07 | 39.37 | 37.10 | 39.21 |
| ChatGPT | 30.62 | 13.70 | 21.33 | 62.26 | 38.30 | 35.38 | 39.46 |
| GPT-4 | 39.33 | 17.75 | 25.54 | 61.97 | 41.59 | 42.62 | 43.43 |

**Table S10.** Results of the summarization models on the SUBJECTIVE division, test set 3. Similar to test 1, the best overall model proved to be $\text{BART+FT}_{\text{SAMSum}}$. In this random sample, the $\text{train}_{\text{UMLS}}$ model was competitive with some of the OpenAI baselines.

| Model | Evaluation score on the OBJECTIVE_EXAM division | | | | | | |
|---|---|---|---|---|---|---|---|
| | ROUGE-1 | ROUGE-2 | ROUGE-L | BERTScore | BLEURT | MEDCON | Average |
| **Retrieval-based** | | | | | | | |
| $\text{train}_{\text{UMLS}}$ | 47.92 | **31.98** | **41.42** | **75.01** | 43.77 | 28.67 | 46.97 |
| $\text{train}_{\text{sent}}$ | 38.01 | 22.79 | 32.66 | 72.09 | 39.05 | 20.46 | 40.69 |
| **BART-based** | | | | | | | |
| BART | 0.00 | 0.00 | 0.00 | 0.00 | 14.95 | 0.00 | 3.74 |
| BART (Division) | 45.55 | 27.45 | 33.89 | 71.52 | 42.17 | 30.91 | 45.06 |
| $\text{BART+FT}_{\text{SAMSum}}$ | 4.42 | 1.25 | 4.10 | 42.16 | 15.05 | 0.83 | 15.33 |
| $\text{BART+FT}_{\text{SAMSum}}$ (Division) | 46.58 | 26.46 | 36.36 | 72.87 | 42.23 | 29.28 | 45.21 |
| BioBART | 5.63 | 1.96 | 5.27 | 43.58 | 15.88 | 2.29 | 16.51 |
| BioBART (Division) | 40.95 | 25.42 | 31.80 | 69.95 | 39.84 | 27.50 | 42.50 |
| **LED-based** | | | | | | | |
| LED | 0.00 | 0.00 | 0.00 | 0.00 | 14.95 | 0.00 | 3.74 |
| LED (Division) | 27.38 | 8.98 | 16.23 | 54.19 | 16.18 | 19.76 | 26.91 |
| $\text{LED+FT}_{\text{PubMed}}$ | 0.00 | 0.00 | 0.00 | 0.00 | 14.95 | 0.00 | 3.74 |
| $\text{LED+FT}_{\text{PubMed}}$ (Division) | 20.16 | 6.13 | 12.10 | 53.62 | 12.84 | 16.44 | 23.93 |
| **OpenAI (wo FT)** | | | | | | | |
| Text-Davinci-002 | 41.63 | 23.33 | 33.26 | 67.47 | 45.79 | 36.25 | 45.56 |
| Text-Davinci-003 | **49.39** | 29.39 | 41.01 | 70.49 | **48.96** | **46.24** | **51.40** |
| ChatGPT | 44.06 | 23.72 | 34.92 | 68.05 | 47.22 | 40.89 | 47.60 |
| GPT-4 | 44.20 | 24.07 | 36.64 | 68.58 | 47.87 | 39.36 | 47.70 |

**Table S11.** Results of the summarization models on the OBJECTIVE_EXAM division, test set 3. Similar to test 1, LED models found this division challenging to summarize with full note and the most performant model based on the averaged score is Text-davinci-003. In this sample, $\text{BART+FT}_{\text{SAMSum}}$ achieved second best performances.

| Model | Evaluation score on the OBJECTIVE_RESULTS division | | | | | | |
|---|---|---|---|---|---|---|---|
| | ROUGE-1 | ROUGE-2 | ROUGE-L | BERTScore | BLEURT | MEDCON | Average |
| **Retrieval-based** | | | | | | | |
| $train_{UMLS}$ | **37.30** | 18.03 | **35.82** | 69.97 | 42.77 | 14.14 | 39.31 |
| $train_{sent}$ | 35.33 | 12.33 | 34.28 | **70.50** | 41.06 | 5.73 | 36.15 |
| **BART-based** | | | | | | | |
| BART | 0.00 | 0.00 | 0.00 | 0.00 | 7.57 | 0.00 | 1.89 |
| BART (Division) | 25.97 | 16.01 | 23.19 | 62.86 | 39.77 | 19.60 | 35.99 |
| BART+$FT_{SAMSum}$ | 33.43 | 0.26 | 33.02 | 61.44 | 39.09 | 0.59 | 30.84 |
| BART+$FT_{SAMSum}$ (Division) | 28.98 | 14.99 | 26.65 | 64.80 | 41.66 | 17.09 | 36.77 |
| BioBART | 32.80 | 0.21 | 32.80 | 60.93 | 38.95 | 0.00 | 30.45 |
| BioBART (Division) | 31.19 | 12.88 | 28.91 | 65.76 | 45.94 | 14.83 | 37.71 |
| **LED-based** | | | | | | | |
| LED | 0.00 | 0.00 | 0.00 | 0.00 | 7.57 | 0.00 | 1.89 |
| LED (Division) | 12.73 | 4.19 | 9.20 | 48.87 | 10.80 | 6.61 | 18.75 |
| LED+$FT_{PubMed}$ | 0.00 | 0.00 | 0.00 | 0.00 | 7.57 | 0.00 | 1.89 |
| LED+$FT_{PubMed}$ (Division) | 9.96 | 3.65 | 7.82 | 41.39 | 5.97 | 6.73 | 15.31 |
| **OpenAI (wo FT)** | | | | | | | |
| Text-Davinci-002 | 30.19 | 16.39 | 28.18 | 63.93 | 44.02 | 15.39 | 37.07 |
| Text-Davinci-003 | 36.83 | **21.14** | 34.66 | 67.38 | **50.28** | 19.92 | **42.11** |
| ChatGPT | 30.66 | 12.92 | 27.89 | 63.72 | 45.31 | 15.36 | 37.05 |
| GPT-4 | 32.63 | 18.57 | 30.21 | 63.88 | 45.68 | **22.53** | 39.81 |

**Table S12.** Results of the summarization models on the OBJECTIVE_RESULTS division, test set 3. Similar OBJECTIVE_EXAM results, LED models performed suboptimally for the objective_results division. This could be as a result of minimal information content for this division (often empty) as well as this content appearing later in a long sequence. OpenAI models performed the best in this division.

| Model | Evaluation score on the ASSESSMENT_AND_PLAN division | | | | | | |
|---|---|---|---|---|---|---|---|
| | ROUGE-1 | ROUGE-2 | ROUGE-L | BERTScore | BLEURT | MEDCON | Average |
| **Retrieval-based** | | | | | | | |
| $train_{UMLS}$ | **47.13** | **23.72** | **31.41** | **71.52** | 44.98 | 28.93 | **44.88** |
| $train_{sent}$ | 39.67 | 17.10 | 25.90 | 67.59 | 42.48 | 16.22 | 38.46 |
| **BART-based** | | | | | | | |
| BART | 0.00 | 0.00 | 0.00 | 0.00 | 32.03 | 0.00 | 8.01 |
| BART (Division) | 43.99 | 19.44 | 25.89 | 66.83 | 42.30 | 31.98 | 42.72 |
| BART+$FT_{SAMSum}$ | 1.47 | 0.61 | 1.12 | 35.59 | 22.28 | 0.26 | 14.80 |
| BART+$FT_{SAMSum}$ (Division) | 43.29 | 19.65 | 26.20 | 67.11 | 42.40 | 34.40 | 43.41 |
| BioBART | 1.10 | 0.78 | 1.01 | 35.32 | 21.72 | 0.85 | 14.71 |
| BioBART (Division) | 44.23 | 20.89 | 27.55 | 67.89 | 43.64 | 31.92 | 43.59 |
| **LED-based** | | | | | | | |
| LED | 0.00 | 0.00 | 0.00 | 0.00 | 32.03 | 0.00 | 8.01 |
| LED (Division) | 28.53 | 5.57 | 12.36 | 55.66 | 27.74 | 20.72 | 29.90 |
| LED+$FT_{PubMed}$ | 0.00 | 0.00 | 0.00 | 0.00 | 32.03 | 0.00 | 8.01 |
| LED+$FT_{PubMed}$ (Division) | 29.37 | 7.02 | 13.34 | 56.15 | 22.73 | 25.56 | 30.25 |
| **OpenAI (wo FT)** | | | | | | | |
| Text-Davinci-002 | 29.70 | 12.72 | 21.59 | 61.40 | 47.26 | 32.82 | 40.70 |
| Text-Davinci-003 | 31.83 | 14.01 | 23.79 | 61.94 | 47.74 | **41.10** | 43.50 |
| ChatGPT | 35.98 | 14.09 | 23.46 | 62.26 | 48.43 | 39.53 | 43.68 |
| GPT-4 | 38.63 | 14.11 | 23.95 | 63.81 | **49.30** | 39.48 | 44.54 |

**Table S13.** Results of the summarization models on the ASSESSMENT_AND_PLAN division, test set 3. BART and LED models trained for full note generation perform suboptimally, likely as this content appearing later in a long sequence. Similar to test 1, the best models for ASSESSMENT_AND_PLAN are the BART Division models and the OpenAI Text-davinci-003 and GPT4 models.
